# Discrete Indoor Three-Dimensional Localization System Based on Neural Networks Using Visible Light Communication

**DOI:** 10.3390/s18041040

**Published:** 2018-03-30

**Authors:** Itziar Alonso-González, David Sánchez-Rodríguez, Carlos Ley-Bosch, Miguel A. Quintana-Suárez

**Affiliations:** 1 Institute for Technological Development and Innovation in Communications, University of Las Palmas de Gran Canaria, Campus Universitario de Tafira, 35017 Las Palmas de Gran Canaria, Spain; itziar.alonso@ulpgc.es (I.A.-G.); carlos.ley@ulpgc.es (C.L.-B.); 2 Telematic Engineering Department, University of Las Palmas de Gran Canaria, Campus Universitario de Tafira, 35017 Las Palmas de Gran Canaria, Spain; mangel.quintana@ulpgc.es

**Keywords:** indoor localization, neural network, visible light communication, received signal strength

## Abstract

Indoor localization estimation has become an attractive research topic due to growing interest in location-aware services. Many research works have proposed solving this problem by using wireless communication systems based on radiofrequency. Nevertheless, those approaches usually deliver an accuracy of up to two metres, since they are hindered by multipath propagation. On the other hand, in the last few years, the increasing use of light-emitting diodes in illumination systems has provided the emergence of Visible Light Communication technologies, in which data communication is performed by transmitting through the visible band of the electromagnetic spectrum. This brings a brand new approach to high accuracy indoor positioning because this kind of network is not affected by electromagnetic interferences and the received optical power is more stable than radio signals. Our research focus on to propose a fingerprinting indoor positioning estimation system based on neural networks to predict the device position in a 3D environment. Neural networks are an effective classification and predictive method. The localization system is built using a dataset of received signal strength coming from a grid of different points. From the these values, the position in Cartesian coordinates (x,y,z) is estimated. The use of three neural networks is proposed in this work, where each network is responsible for estimating the position by each axis. Experimental results indicate that the proposed system leads to substantial improvements to accuracy over the widely-used traditional fingerprinting methods, yielding an accuracy above 99% and an average error distance of 0.4 mm.

## 1. Introduction

The Global Positioning System (GPS) is the best known satellite-based navigation system which has been applied into various location-based services. However, the GPS system does not work well for indoor environments because it does not have lines-of-sight (LOS) for signal transmissions from satellite, and therefore, it has a low accuracy [1]. Indoor localization has gained considerable attention over the past decade due to the emergence of numerous location-aware services. These new services have made it possible to develop applications capable of sensing location to offer services or dynamically adjusting their settings and functions [2]. In fact, many positioning systems based on radiofrequency communication systems, such as WiFi [3,4], Bluetooth [5], Zigbee [6] or RFID [7] have been proposed, mainly for having been globally deployed and for their low cost. Nevertheless, multipath fading causes the received signal to fluctuate around a mean value at a particular location [8]. Hence, those systems usually deliver an accuracy of above two metres, and could not be suitable for environments with high accuracy requirements. Thus, precise indoor localization is a still critical missing component which has gained a growing interest from a wide range of location based applications, such as robotics, tracking of disabled people, etc.

On the other hand, optical wireless communications based on visible light [9], named Visible Light Communications (VLC), are used to transmit data by modulating intensity in light emitting diodes (LED), employing faster switching rates than the persistence of the human eye to avoid flickering in data/light sources. The increasing use of LEDs in illumination systems has conducted the emergence of the VLC technologies, both in indoor and outdoor environments [10]. In indoor location applications, VLC allow for a greater precision than outdoors.

In recent years, positioning systems based on VLC have been an attractive research topic. VLC can offer high accuracy in positioning, mainly due to the fact that this kind of networks is not affected by electromagnetic interference and because the optical signal is more stable than radio frequency signals. Indoor localization systems using LEDs have shown to be more accurate with 0.1–0.35 m positioning error when compared to WiFi (1–7 m) and Bluetooth (2–5 m) [11]. Thus, in this manuscript a discrete indoor localization system using a VLC infrastructure and based on three neural networks is proposed. Each neural network is responsible for infering a value of *X*, *Y* or *Z* axes and thus the coordinate of device location is estimated. RSS values from the transmitters located in the ceiling are used as input in each neural networks. To evaluate the effectiveness of the proposed model, a dataset has been created considering LOS and reflections. The dataset also includes variations in the tilt of angle of the receivers with regard to the transmitters. The simulation results yield a 99.80% accuracy and an average error of 0.4 mm.

The paper is organized as follows: Section 2 summarizes the related work about indoor localization using VLC. Section 3 explains the optical channel model and the characteristics of simulation scenarios used to generate the RSS dataset using CandLES tool. Next, in Section 4, the location system based on neural networks is described. In Section 5, the results are discussed and the performance and robustness are analysed. Finally, in Section 6 the conclusions and future work are presented.

## 2. Related Work

Recently, authors in [12] mainly classified VLC based positioning techniques into four groups: proximity, fingerprinting, triangulation and vision analysis. In the proximity method, the location of the device is determined on the basis the signal coming from a single LED base station. Each LED base station has an identifier code. When a device receives the signal from a LED base station, it also receives its identifier code. The receiver has a database that associates IDs with locations. The position of mobile device is determined as the whole area covered by the light radiated from the LED whose ID is received by the device. This technique cannot give absolute or relative positions but only proximity location information.

On the other hand, the fingerprinting technique estimates the position by matching online measured data with pre-measured location-related data, such as RSS. Localization based on fingerprinting is usually carried out in two phases. In the first phase, normally termed the offline phase, a database of the RSS samples is built from different base stations at each reference location for the target environment. With the samples as training set, a position model is learned using a particular machine learning technique. During the second phase, online phase, the location is determined by means of new RSS measurements collected in a particular position and the built model.

In the third technique, triangulation, the position is calculated by using properties of triangles, based on geometrical properties: latitude and angulation. The lateration method estimates the target position, by measuring distances from the receiver to multiple LEDs that work as access points. The positions of these access points are known. The distances can be estimated by the time of arrival (TOA), time difference of arrival (TDOA) and the RSS. In the second method, called angulation or angle of arrival (AOA), the target position is estimated by measuring the angles to multiple base stations or access points. Frequently, all these techniques require additional hardware, time synchronization between emitters and receivers, being necessary to know the coordinates of the access points and also demand computational cost.

Finally, the technique of vision analysis relates geometrically 3D positions of objects with their 2D projection on an image sensor. Geometric relationships are obtained with a pinhole camera model.

Nowadays, the indoor localization based on fingerprinting is one of the most used technique [13]. The simplest system only needs RSS information and additional sensors are not needed. Many researchers apply new machine learning prediction techniques in order to get best results. Thus, neural networks are an effective classification in machine learning and can be applied for positioning estimation. Neural networks are trained to recognize a set of patterns. Some research apply neural networks as a fingerprinting positioning method. Authors in [6] employed two feed-forward neural networks using three fully connected layers and trained with the back propagation algorithm. One of the networks estimates the Cartesian coordinates and the other one estimates the polar coordinates. RSS values from Zigbee wireless sensors are used as input to the networks. Also, authors in [14] proposed the use of two multilayer neural networks trained independently to estimate the (x,y), coordinates from WiFi RSS. Furthermore, the use of a multilayer neural network using a back propagation algorithm to estimate the coordinates (x,y) is proposed in [15]. Contrary to other jobs, the inputs to the neural network are normalized RSS samples and standardized multipath parameters from several access points are used as input to the neural network. All these works are focused on the estimation of the coordinates in the bidimentional plane using communication systems based on radiofrequency.

On the other hand, some research works estimate three-dimensional coordinates using a visible light communication infrastructure. These works are based on estimating the position from RSS samples, TDOA technique and the application of the algorithms for solving equations. In [16] a three-dimensional positioning scheme for indoor VLC systems is proposed. Precise location estimation of the terminal device can be achieved by measuring RSS through LOS channels. The average error is about 2.5 mm. In order to get this accuracy, the mobile device has multiple PD (Photodiodes), receivers, where relative positions have to be known. This system uses all the RSS of all PDs to estimate the position. Authors in [17] proposed a wireless accurate three-dimensional localization system using white LED lights, using frequency division multiplexing (FDM) and the TDOA technique for 3D localization. The average error is very small, below 1 mm, but the experiments were made with all receivers located on the same plane. The authors measure phase differences among the received signals with respect to a reference LED that along with a positioning algorithm returns the coordinates of location by solving a set of linear equations. In [18] a three-dimensional positioning algorithm is also proposed using RSS, which changes according to the angle and distance between transmitters and receivers. The accuracy of this system is about 3 cm, but to estimate the position, the system requires other data or parameters as the tilting, and also the application of an angle compensation algorithm in addition to the received signal RSS. At last, a genetic algorithm to solve the positioning problem using a VLC network is described in [19], yielding an average error of 5.55 cm. To estimate the coordinates, each LED transmitter has an ID, combined with intensity attenuation information and CDMA modulation.

There is no doubt that significant progress has been made in the field of indoor localization using VLC networks. However, an improvement to the actual approaches is needed, giving a better and precise three-dimensional indoor positioning. The main novelty of this work comes from the fact of applying neural networks as a predictive technique for three-dimensional positioning systems. That is, the proposed system processes a grid of received signal strength (RSS) to estimate the Cartesian coordinates (x,y,z) of the mobile device. To the best knowledge of the authors, the application of neural networks to the positioning problem has been just implemented in radiofrequency environments and only estimate coordinates on a plane. This work includes the experiments of working with tridimensional positions, for which the combination of three neural networks is used, where each of them is responsible for estimating positions within the *X*-axis, *Y*-axis and *Z*-axis respectively. The positioning system proposed in this article has an accuracy of above 99% with an average error distance of 0.4 mm, using only the power received signals as input to the neural networks. In addition, it is a low complexity system, therefore it is suitable for integration into mobile devices.

## 3. Simulation Model

In a VLC positioning system, the characteristics of the LEDs, transmitters, the PD receivers, as well as the channel model must be taken into account. In this section, basic aspects of the channel model and transfer function of these systems are given.

### 3.1. Channel Model in VLC

**Figure 1:**
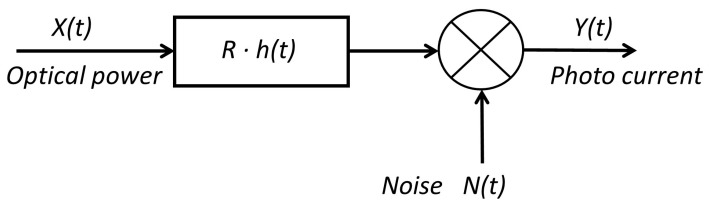
Optical channel modeled as a base band linear system.

The optical channel components are the followings: optical transmitter (LED), photo detector (PD) and transmission medium. For VLC links, intensity modulation (IM) is used, in which the waveform of the signal to be transmitted is modulated onto the instantaneous power of the optical carrier. The technique used in reception is direct detection (DD), in which a photo detector produces an electrical current proportional to the received optical instantaneous power. Usually, optical wireless systems based on IM/DD are modeled as a base band linear, time-invariant system [20], see Figure 1.

In Figure 1, X(t) is the instantaneous input power, Y(t) is the output current, and h(t) is the impulse response. N(t) is the signal-independent additive noise and *R* is the receiver responsivity. This base band channel model can be expressed by Equation (1), where ⊗ symbol denotes the convolution operation.

(1) Y(t)=R·X(t)⊗h(t)+N(t)

The impulsive response, h(t), is determined by transmitter and receiver characteristics, but also depends on their position, orientation and optical signal reflections as well. Plenty of works have been published to characterize optical wireless channel and its impulsive response h(t), such as [21] based on evaluating of measures, Lopez-Hernandez et al. [22] which applies iterative algorithms, or [23] based on statistical methods. Others works have been also published focusing on studying the VLC channel, such as [24,25,26].

**Figure 2:**
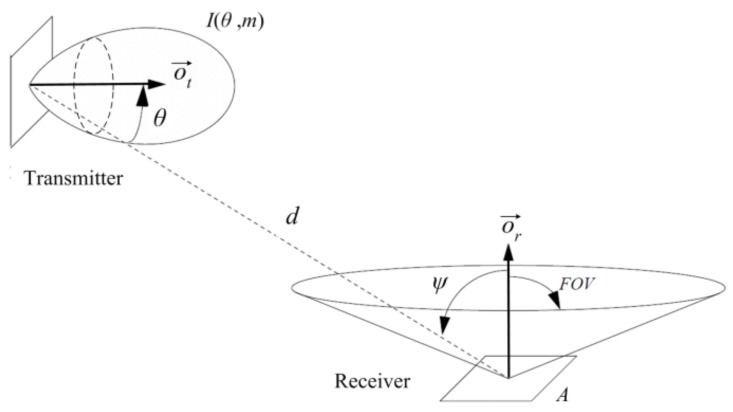
Transmitter and receiver in directed LOS link configuration.

In VLC, the received power can be expressed as the sum of LOS and non line of sight (NLOS) components [26]. In directed LOS links, the h(t), hence the DC gain can be computed fairly accurately by considering only the direct LOS propagation path. Figure 2 shows an example of a directed LOS link. An optical source can be modeled by its position vector, a unit-length orientation vector ${\vec{o}}_{t}$, transmission power $P_{t}$ and a radiation intensity pattern I(θ,m) emitted in direction θ. Here *m* is the mode number of the radiation lobe, which specifies the directionality of the source, and is related to the transmitter half power angle $\theta_{1/2}$. Similarly, a receiver is defined by its position, orientation ${\vec{o}}_{r}$, the photo detector area *A*, and the field of view (FOV). The angle formed between the optical incident signal and the orientation vector ${\vec{o}}_{r}$ is called the incident angle ψ. The maximum incident angle defines the receiver FOV.

According to [20], when considering only the direct LOS propagation path, the channel DC gain *H*(0) is given by Equation (2), where T(ψ) is the signal transmission coefficient of the optical filter in receiver, G(ψ) is the receiver optical concentrator gain, and *d* is the distance between transmitter and receiver. Equation (2) is based on considering the optical transmitter as a single point source, though VLC transmitters tend to be composed by a large LED array in order to improve illumination capacity.

(2) $$H\left(0\right)=\frac{P_{r}}{P_{t}}=\left\{\begin{array}{cc} \frac{m+1}{2\pi \cdot d^{2}}\cdot \cos^{m}\left(\theta \right)\cdot A\cdot G\left(\psi \right)\cdot T\left(\psi \right)\cdot \cos\left(\psi \right) & 0<\psi \leq FOV\\ 0 & \psi >FOV \end{array}\right\}$$

Authors in [27] compared the channel characteristics of both the single point-source model and the array of LEDs. The results obtained show that the deviations are acceptable in terms of the channel optical path loss, as well as bandwidth. There are differences in terms of RMS delay-spread results, though they remain acceptable as long as the LED array is of moderate size.

### 3.2. Simulation Software: CandLES

In order to validate the proposed system in this work, a RSS dataset was generated using CandLES software [28]. CandLES is a Communication and Lighting Emulation Software that uses MATLAB. This tool has modulation, transmitters, optics, channel, noise, interference, receivers and decoding components. It allows us to fix parameters such as room sizes, objects, orientation, shadowing, and wall reflectivity. In order to calculate the channel impulse response, h(t), and the received power, $P_{r}$, CandLES adopts a fast algorithm developed for IR free space optical communications [29]. This model takes into account: locations of transmitters, receivers, obstacles, reflectivity of each wall and obstacle, field of view of receivers, receiver area and the number of reflections.

Thereupon, the most important features of our model are described, which are needed to adequately interpret the results obtained from the simulations performed. We selected commonly used values to characterize VLC transmitters and receivers, similar to those used in [30,31,32]. Optical transmission power of devices is of 15 W. According to the optical channel model used, transmitters’ directivity is characterized by its half power angle, $\theta_{1/2}$, while receivers’ directivity is defined by its FOV. According to [25], both parameters are assigned a value of 60°. The transmission medium is modeled as free space without obstacles. In order to calculate RSS values the direct component of the received signal and the existence of reflections are considered. All optical receivers have been configured using the value of 60° for FOV, a photo sensor area of 100 mm^2^, an optical concentrator gain of 10, and a concentrator refractive index of 2.73. Finally, the reflectivity (%) of wall, ceiling and floor were set to 0.58, 0.69 and 0.09, respectively.

## 4. Localization System Based on Neural Networks

**Figure 3:**
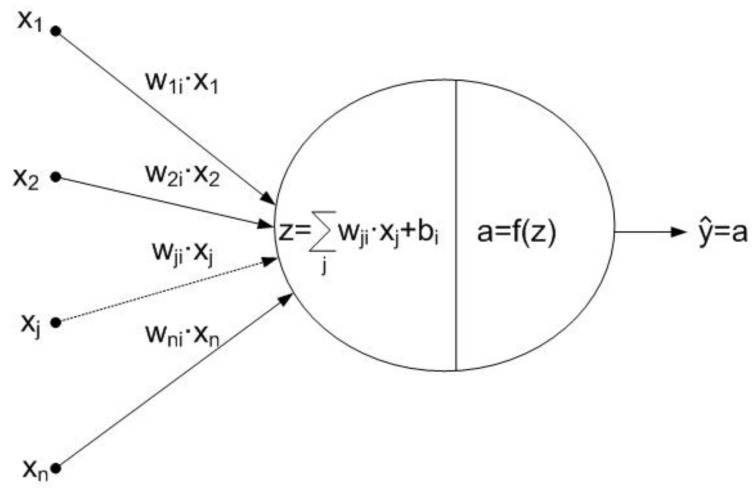
Node basic structure.

**Figure 4:**
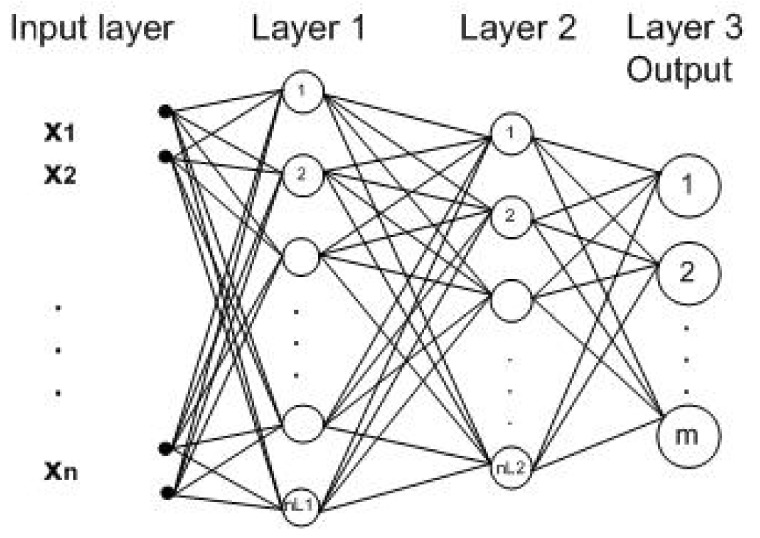
Neural network basic structure using two hidden layers.

In this section, a discrete indoor localization system using three neural networks is described. Neural networks have proved to be very effective and powerful tools in solving problems of classification and prediction in maching learning, and hence can be trained to recognize any set of input patterns by predicting an output. A neural network consists of an input layer of nodes, one or more hidden layers and one output layer. The nodes in hidden layers are fully connected, and each connection has a weight $w_{ji}$ and bias $b_{i}$. The basic structure of a node is shown in Figure 3, and the basic structure of a neural network is shown in Figure 4.

The output of each node is given by Equation (3), where $x_{j}$ are the inputs to the node, and f(·) is the transfer function such as *sigmoid*, *tanh* or *lineal* function.

(3) $$\hat{y_{i}}=f\left(\sum_{j}w_{ji}\cdot x_{j}+b_{i}\right)$$

The learning of a neural network consists of minimizing a cost function, Equation (4), which is a function that depends on the weights, *w*, and bias, *b*, of the neural network, where $\hat{y}$ is the estimated output and *y* is the target output used to train the system.

(4) $$J\left(\hat{y},y\right)=f\left(w,b\right)$$

There are different algorithms to train a neural network, but in this work a Matlab implementation of the Scaled Conjugate Gradient algorithm was used [33]. During the training process, the weights are adapted in order to minimize the cost function. The training phase can end when the cost function reaches a minimum or when the number of iterations reaches a given value.

Our work focuses on the use of a modular system of neural networks, the well known Multilayer Perceptron (MLP) architecture. This neural network has been used in a variety of ways and applications. As it is a three dimensional positioning system, the problem is broken down into three neural networks, one per axis, *X*-axis, *Y*-axis and *Z*-axis, where the output node of each neural network determines a position on the *X*-axis, *Y*-axis or *Z*-axis. In fact, Sharkey in [34] said that modular decomposition can be undertaken for the purpose of improving performance. The “divide and conquer” principle, whereby the task is divided into a number of sub-problems can be used to extend the capabilities of a single net. Each subtask could then be solved with a different neural net architecture or algorithm, making it possible to exploit specialist capabilities. Another reason for adopting this approach is that of reducing model complexity and computational cost, making the overall system easier to understand and extend.

In order to model the neural networks, an array of RSS samples for each receiver is used as input to the system. This array, *X*, has $n_{Tx}\times m_{x}$ dimension, where $n_{Tx}$ is the number of transmitters or LED lamps, and $m_{x}$ is the number of RSS samples. However, the output of each neural network is different from the rest, and it depends on the receivers’ distribution.

For *X*-axis and *Y*-axis, the output arrays, $Y_{x}$ and $Y_{y}$, have a dimension equal to *number of receivers per row or column*. For *Z*-axis, the $Y_{z}$ array has a dimension equal to *number of planes*.

Therefore, the set pair of (X,Y) is obtained for each of the neural networks, where $X\in R^{n_{Tx}\times m_{x}}$, $Y_{x}\in {\left(0,1\right)}^{receiversByRow\times m_{x}}$, $Y_{y}\in {\left(0,1\right)}^{receiversByColumn\times m_{x}}$ and $Y_{z}\in {\left(0,1\right)}^{planesNumber\times m_{x}}$

## 5. Results and Discussion

In this section, the RSS dataset from a VLC network and the results obtained from experiments carried out to evaluate the best configuration and efectiveness of the proposed system are described and discussed. Experiments were focused onto comparing accuracy and error distance varying the number of nodes in each hidden layer, and the robustness and computation time with different training sizes. The error is the expected distance from the misclassified instance (estimated receiver) and the real location (real receiver). This error is obtained by calculating the Euclidean distance between these points, and the arithmetic mean was computed from the results of the experiments.

The dataset was randomly divided into training, validation and test sets, with a size of 70%, 15%, and 15% of RSS samples from whole dataset, respectively. The training set is used to train the network. Training continues as long as the network keeps improving on the validation set. The test set provides a completely independent measure of network accuracy. In order to validate the experimental results, and to ensure statistical independence, all experiments have been repeated 100 times. The system was implemented using Neural Network Toolbox of Matlab. All experiments were carried out on an Intel Core i7 3.4 GHz/32 GB RAM non-dedicated Windows machine.

### 5.1. Dataset

**Figure 5:**
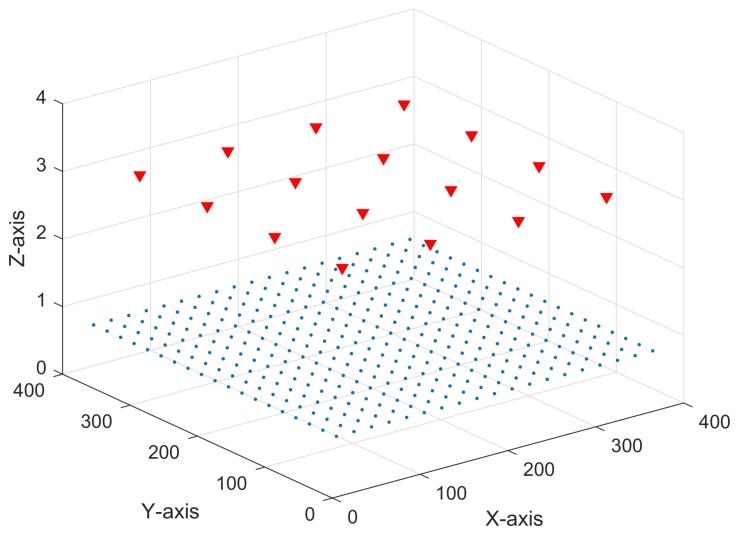
Grid of receivers at 0.75 m from the floor.

**Figure 6:**
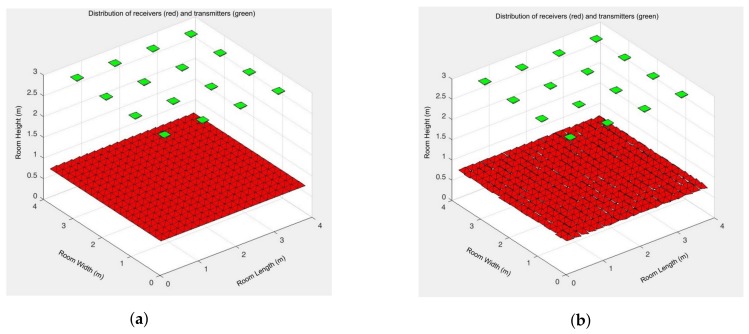
Receivers orientation: (**a**) each receiver points towards the ceiling with 90°; (**b**) each receiver points towards the ceiling with a randomly angle.

As was aforementioned, RSS samples are used to model the neural networks proposed in this work. For that, CandLES software was used to build a RSS dataset where both direct component and reflections of the optical transmissions were taken into account in a 4 by 4 by 3 m room. Figure 5 illustrates the simulation scenario. This environment consists of 16 LED lamps or transmitters (red triangles), configured as a 4 × 4 grid placed 1 m apart from each other on the ceiling. On the lower part, we set up 361 receivers (blue circles) in a 19 × 19 grid configuration, with a 20 cm separation from each other. In order to evaluate the effects of having different distances between receivers and transmitters, the receivers plane is set up at three different heights: 75, 100 and 125 cm from the floor. The following simulations were carried out to generate the dataset:

- *Direct component.* 11 simulations taking into account only the direct component were carried out. All receivers pointed out to the ceiling with at a 90 degree angle in the first simulation, Figure 6a. The rest of the simulations were done with the receivers pointing towards the ceiling with different random angles, between [−105°, +105°], Figure 6b. Thus, each receiver has a different orientation in each simulation.
- *One reflection.* 11 simulations taking into account the first reflection were carried out, as done in the previous case.
- *Two reflections.* 11 simulations taking into account two reflections were carried out, as done in the previous case.
- *Three reflections.* 11 simulations taking into account three reflections were carried out, as done in the previous case.

Therefore, forty four simulations were performed on each one of the three aforementioned receivers planes, 75 cm, 100 cm, and 125 cm. One RSS measurement from each LED lamp was estimated at each receiver in every simulation. This leads to 17,328 (16 LED lamps × 361 receivers × 3 layers) RSS measurements in each simulation. Hence, the whole dataset is composed by 762,432 (17,328 × 44 simulations) RSS measurements. The simulation parameters were specified in Section 3.2.

**Figure 7:**
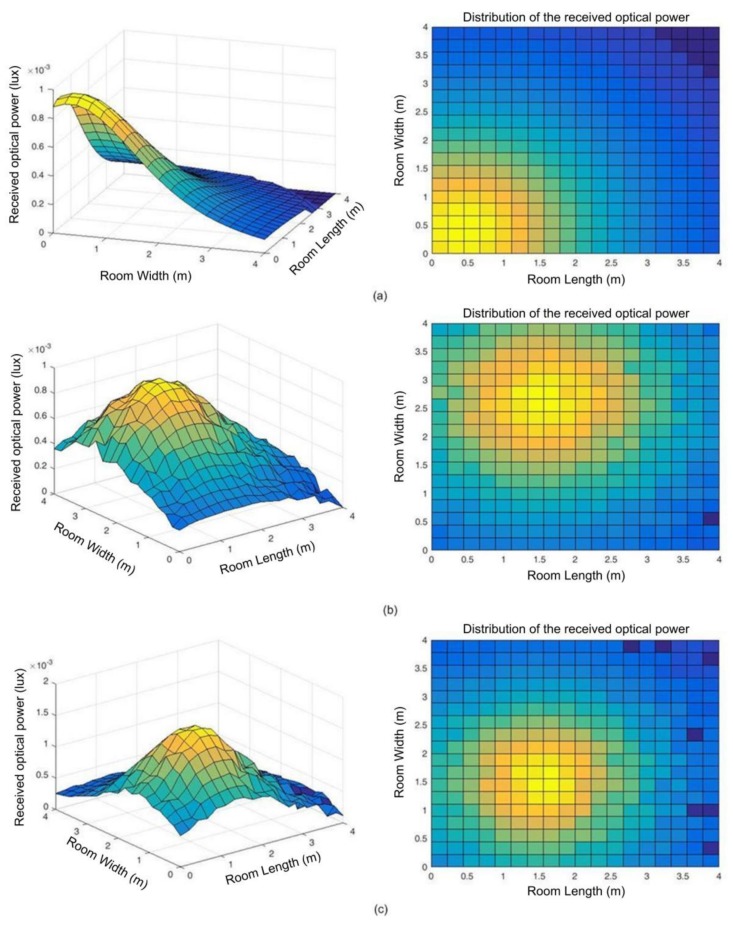
Examples of power distribution: (**a**) LED 1 is powered on and all receivers are pointed out to the ceiling; (**b**) LED 10 is powered on and all receivers pointed out with different angles; (**c**) LED 6 is powered on and all receivers pointed out with random angles and three reflections are taken into account at receivers.

Figure 7 shows the received optical power (lux) at 1 m from the floor when only one transmitter is powered on. The first image (a) is an example showing when the transmitter number 1 is powered on and there are no reflections, thus only the direct component of transmission is detected by the receivers. All the receivers are pointing out to the ceiling with 90 degrees. Figure 7b shows the power distribution when only the transmitter number 10 is powered on and the receivers point out to the ceiling with different angles. Finally, Figure 7c shows a representation when LED 6 is powered on, the receivers are configurated with random pointing angles taking into account up to three reflections.

### 5.2. Neural Network Configuration

**Figure 8:**
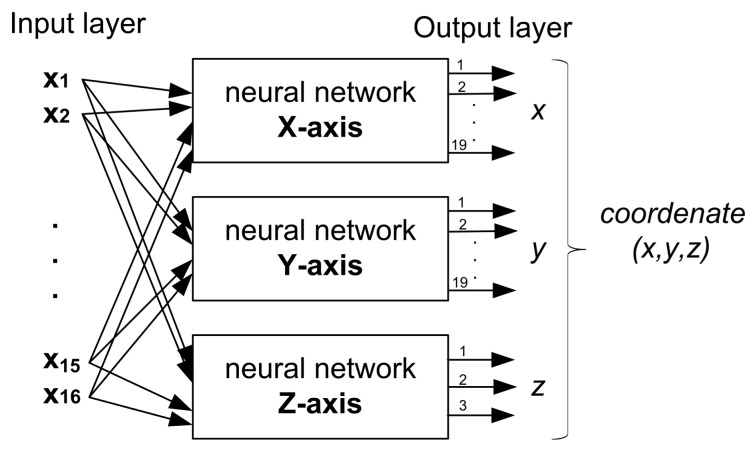
Proposed system based on three neural networks where each of them estimates the position in the axes *X*, *Y*, and *Z*.

Due to there are 16 LED lamps, the input layer of each neural network has 16 nodes. In addition, from dataset, each receiver $Rx_{i}$ of the simulation environment has a fixed position ${\left(x,y,z\right)}_{i}$ where 1<i<361. There are 19 possible locations in the *X*-axis, 19 locations in the *Y*-axis and 3 locations in the *Z*-axis. This clearly defines the output layer of the neural networks of these axes. Therefore, the neural networks that define the *X*-axis and the *Y*-axis will have 19 nodes in their output layer. For the *Z*-axis, three different heights were considered, the receivers were placed on planes of 75 cm, 100 cm and 125 cm from the floor. Therefore, to estimate the position in the *Z*-axis, a third neural network was combined with 3 nodes in the output layer, so each node defines a height or plane. Thus, the input and output layers configuration of the proposed three dimensional system in this paper is shown in Figure 8.

On the other hand, a first experiment using a basic structure with only hidden layer was carried out in order to determine the best configuration of the neural networks. However, preliminary results yielded an accuracy of about 80%. Taking into account the propagation characteristics of the optical signal, it is expected to achieve at least a 90% accuracy. Therefore, a second hidden layer was added to each neural network to reach that accuracy.

Using this structure, and in order to find the network architecture with best accuracy, different experiments were carried out by varying the number of nodes in both hidden layers. Concretely, the amount of nodes for the first hidden layer was varied from 10 to 100 in steps of 10. The number of nodes for the second hidden layer was varied from 10 to 50 in steps of 10. Table 1 shows the results in percentages of accuracy for the training dataset. As can be seen, the poorer accuracy is obtained when 10 nodes are used in both layers, although it remains above 90%. The best accuracy, 99.8%, is achieved when 80 and 30 nodes are used in the first and second layers, respectively. Also it is noticeable that when the number of hidden nodes in the second layer is low, more nodes in the first hidden layer are needed to get a high accuracy. Furthermore, as the number of nodes in the second hidden layer increases we need fewer nodes in the first hidden layer to obtain a similar accuracy. Finally, when using 50 nodes in the first layer, an accuracy above 99% is achieved, regardless of the number of nodes in the second layer.

On the other hand, the average error distance was also calculated for all experiments. Table 2 shows the neural network architectures with the lowest average error yielding the best average error distance with 0.39 mm. These architectures match with the highest accuracy rate in Table 1. As can be seen, the neural network architecture with the lowest average error distance is the 80-node neural network for the first layer and 30 nodes for the second layer. This neural network will be taken as reference to assess the model presented in this work.

**Figure 9:**
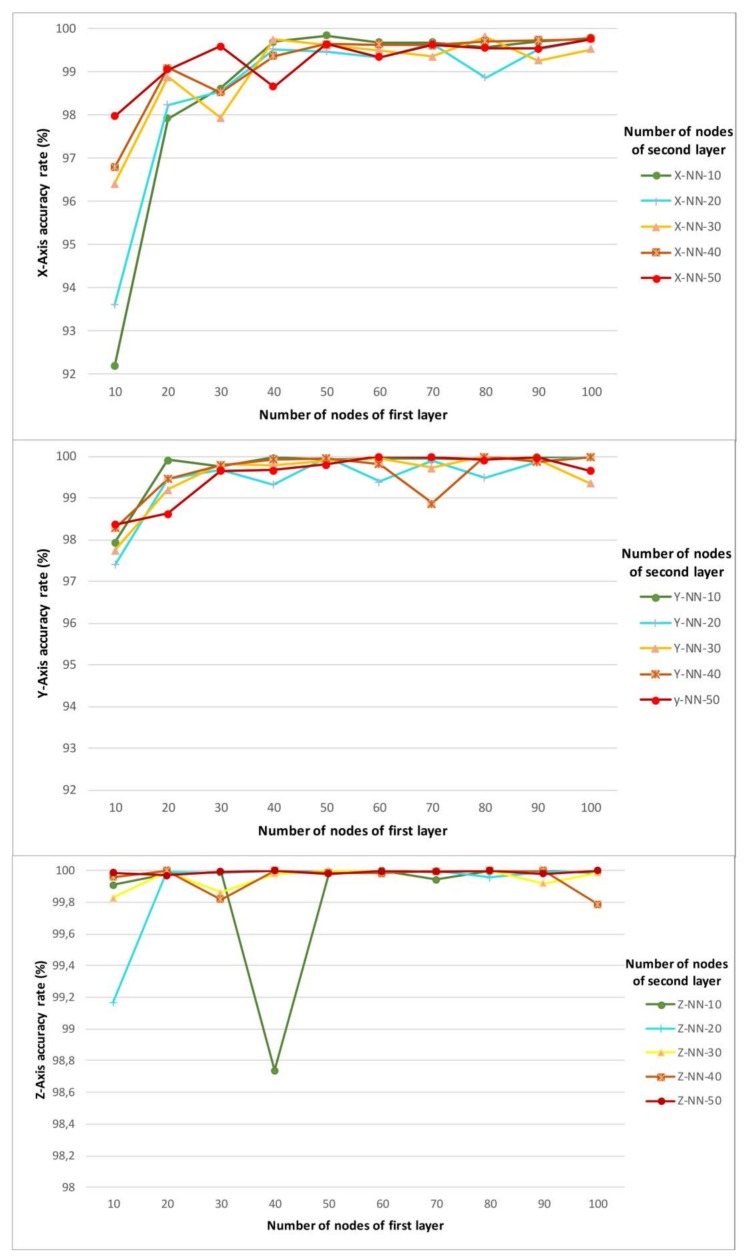
Accuracy rates (%) for each neural network.

Next, the architecture of each neural network is analysed. Figure 9 shows the accuracy rate (%) for each neural network or by axis. These graphs show that the network of the *X*-axis requires a greater number of nodes in the first hidden layer. From 50 nodes in this first layer, the accuracy is practically the same regardless of the nodes of the second layer. The neural network of the *Y*-axis presents a more stable behavior when the number of nodes of the first layer is lower compared to the neural network of the *X*-axis. The neural network of the *Z*-axis has a much more stable accuracy rate. This is mainly because it is a simpler architecture and it only has to estimate 3 classes.

### 5.3. Neural Networks Robustness

In order to validate the robustness of neural networks application for indoor localization, the efficiency of this approach was tested by varying the training dataset size from 15% to 70%. All experiments were performed using the combination of number of nodes in layers with the best accuracy and lower mean error, that is, 80 and 30 nodes in the first and second layers, respectively.

Table 3 shows the experimental results obtained by varying the training size. As can be seen, the system accuracy increases when the training size does yielding excellent results with a low number of training samples. Thus, when the number of training samples is 15% this approach decreases in effectiveness, but even in these circumstances the system gets an accuracy above 97%. For other training sizes, the accuracy of system is above 99%. Furthermore, precision, recall and F-Measure measurements also follow a similar behavior related with accuracy. Thus, the measurements increase with the training size, keeping values close to 0.97 when using only a 15% of training size and close to 0.99 when using a 30% or more of training size. Furthermore, a lower training dataset size offers savings in computational cost and time needed to compute the neural networks. In fact, as shown, using the smallest dataset size, the training time is about 12 times less than the time spent using a 70% dataset size.

**Figure 10:**
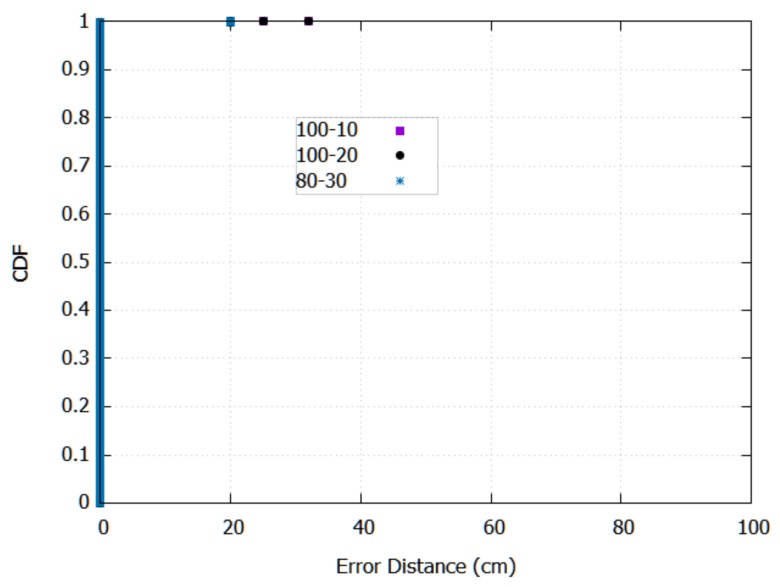
CDF for 100-10, 100-20 and 80-30 nodes.

Finally, Figure 10 shows the cumulative distribution function (CDF) for the best structures of hidden layers. As can be seen, most of the test instances are correctly classified, and most of the misclassified instances are about 20 cm, that is, these instances are the nearest neighbours (receivers) of exact locations in the same height.

### 5.4. Evaluating the Model with Other Dataset

**Figure 11:**
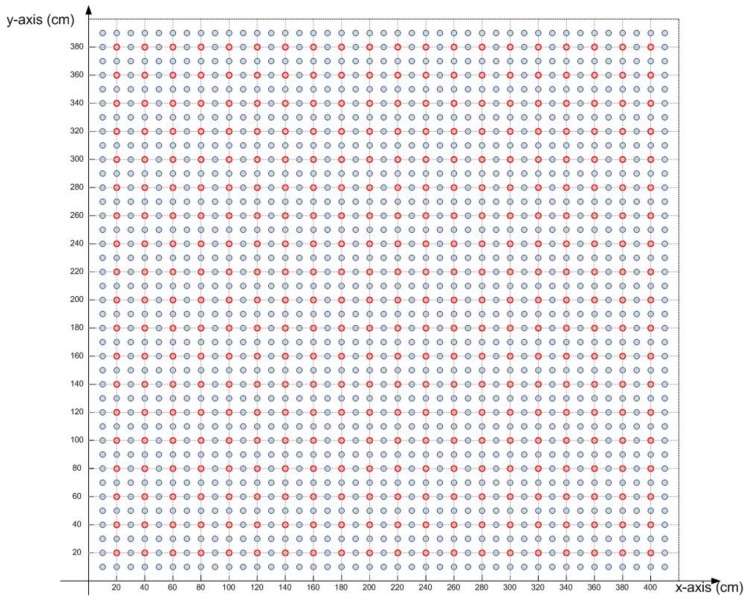
Distribution of receivers in the room in the *xy* plane.

To demonstrate the usefulness of the proposed method, we implement a new dataset of RSS values using CandLES. A new mesh of receivers placed every 10 cm was made. No receiver was placed on the edge of the room. Therefore, the mesh has 39 × 39 receivers, that is 1521 receivers. Figure 11 shows the receivers located every 20 cm in red colour, and the new receivers provided by this dataset are coloured in blue. This experiment was made in order to have new positions different from those used in training and analysing the estimation of coordinates made by the three neural networks.

**Figure 12:**
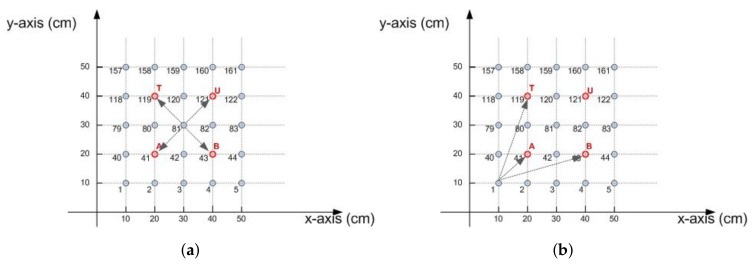
Estimation of positions. (**a**) A receiver is at the same distance from four candidate points of the training mesh; (**b**) Possible estimation errors.

In order to verify the performance of the neural networks for this new dataset, the following considerations were made:

- If the receiver is located at a point in the training dataset, the result of the neural network must estimate the position of training dataset, for example, node 41 of Figure 12a, matches node A of training. In this case the estimated position will be exactly determined, otherwise it will result in error.
- The receiver is located at the same distance from two or more points. For example, in Figure 12b, the node 81, is at the same distance from points A,B,T,U, (points of training mesh). The same thing occurs with the node number 3 from points A,B. In this case, a vector of candidate positions is calculated by measuring the Euclidean distance, Equation (5).
  (5) $$d_{i}=\sqrt{({\left(p_{z}-{\left(n_{z}\right)}_{i}\right)}^{2}+{\left(p_{y}-{\left(n_{y}\right)}_{i}\right)}^{2}+{\left(p_{x}-{\left(n_{x}\right)}_{i}\right)}^{2}}$$
  For receiver number 81, the candidate vector is calculated, see Equation (6). The three neural networks will have made a correct guess if the estimated position is contained within the vector of candidate positions. The estimate will be considered correct.
  (6) $$v_{81}=d_{A},d_{B},d_{T},d_{U}$$

In order to evaluate the performance of the proposed model, the previous considerations were taken into account. The accuracy rate are shown in Table 4. The accuracy rate is about 97% for this dataset. One of the best results is for neural network with 80-40 nodes yielding a 97.38% accuracy and the best distance error, only 9.15 cm, Table 5.

If the training or the parameters of the neural network are not adequate, a few cases can be found where the error distance can be relatively higher than foreseen. It is expected that by the distribution of powers received by the device, the neural network estimates the position of the mesh that fits most position nearby points. If the calculated point moves away from the neighborhood, error in distance may be greater than expected, see Figure 12b.

This is because the training axes are discretized by the training mesh. If only one of the coordinates estimated by one of the three neural networks is wrong, jumping to points outside of the neighborhood causes the increase of this error.

Finally, a comparative study has been done with other state-of-the-art 3D indoor localization techniques using VLC networks. As can be appreciated in Table 6, the results obtained in this work outperform other research works, being able to achieve a low error distance. In [17] the achieved error distance is below than our work but it has a more computational cost because is based on TDOA.

## 6. Conclusions

As was discussed in this manuscript, indoor localization has become an attractive research topic due to growing interest in location-aware services, and several systems have been proposed in the literature. In this paper, a discrete three-dimensional indoor localization system based on neural networks and using the RSS values from LED lamps of a VLC network is proposed. In order to validate the system, the CandLES tool was used to build a RSS dataset taking into account the direct component and multipath reflections of the optical signal. Furthermore, random orientation angles for receivers were also considered. In order to find the best architecture of neural networks several experiments were carried out by varying the number of nodes in the hidden layers. As far as authors know, they are not evidences of other researches that have considered a system with three neural networks for indoor localization. Even so, the experimental results have demonstrated that the proposed system yields a high accuracy, with achieved results for accuracy above of 99% and average error distance about 0.4 mm.

On the other hand, the proposed system achieves an accuracy about of 97% and an average error distance of about 9 cm when tested with a dataset with receivers located every 10 cm. Therefore, and due to being a discrete system, it is expected that most of the errors are the nearest neighbors (receivers) of real locations, that is, just the grid separation. In spite of the discretization and the fact that the error is fixed by the grid separation, due to the high accuracy and the low computational complexity, the proposed system is adequate to be implemented in devices with restricted energy consumption and limited computing power. Finally, the proposed system was analysed using scenarios with reduced training datasets, validating the robustness of the proposed solution. Effectiveness of the system is reduced when the training dataset size decreases, but even so, with only a 15% of the samples for training, this approach yields an accuracy above 97%.

In our ongoing work, we are planning to design and develop a VLC network to validate the proposed system in a real environment. Thus, once three neural networks are trained, the weights and bias can be loaded on low cost devices and therefore the performance and energy consumption of system can be evaluated in real conditions. On the other hand, the proposed approach is a discrete system, where neural networks estimate the position within the grid for which they have been trained. Therefore, we are also planning to apply regression techniques to calculate the exact position of mobile devices using neural networks.

## Figures and Tables

**Table 1:** Accuracy results (%) depending on the number of nodes in hidden layers.

Nodes Layer 1	Nodes Layer 2				
	10	20	30	40	50
**10**	90.4704	90.8356	94.2940	95.2635	96.4303
**20**	97.8154	97.7314	98.1218	98.5708	97.7251
**30**	98.3988	98.2393	97.6517	98.1658	99.2634
**40**	98.4176	97.8751	99.5299	99.3116	99.3400
**50**	99.7607	99.4417	99.5131	99.5760	99.4438
**60**	99.6432	98.7534	99.4459	99.4375	99.3347
**70**	99.5802	99.5551	99.0997	98.5268	**99.6054**
**80**	99.4942	98.3547	**99.8006**	**99.6999**	99.4669
**90**	99.6642	99.3767	99.1312	99.1312	99.4942
**100**	**99.7628**	**99.7250**	98.8898	99.5446	99.4291

**Table 2:** Error distance in cm.

	Nodes Layer 1-Nodes Layer 2				
	100-10	100-20	80-30	80-40	70-50
**Average error**	0.048	0.055	0.039	0.060	0.086

**Table 3:** Performance results depending on training size.

Training Size	Accuracy	Precision	Recall	F-Measure	Training Time (s)
15	97.50	0.9751	0.9751	0.9874	63.46
30	99.09	0.9869	0.9869	0.9934	292.31
50	99.40	0.9941	0.9940	0.9970	580.17
70	99.47	0.9947	0.9947	0.9974	880.84

**Table 4:** Accuracy rate for a new RSS dataset, testing all the trained neural networks.

Nodes Layer 1	Nodes Layer 2				
	10	20	30	40	50
**10**	91.5157	91.4759	93.6241	94.3323	94.4608
**20**	95.5880	95.7962	95.9989	96.1817	95.8988
**30**	95.7010	96.0557	96.0617	95.5979	96.7764
**40**	95.9192	96.2539	96.9468	96.8780	96.1164
**50**	96.3142	97.2022	96.9348	97.1554	96.8073
**60**	96.9497	96.7301	97.2033	96.8675	96.8989
**70**	97.0628	**97.3965**	96.6863	96.9129	96.9622
**80**	96.5000	96.5463	**97.2411**	**97.3816**	96.7794
**90**	**97.3352**	97.2849	96.6783	97.1340	**97.1440**
**100**	97.0205	97.2222	97.2760	97.1514	97.0827

**Table 5:** Error distance in cm.

	Nodes Layer 1-Nodes Layer 2				
	90-10	70-20	80-30	80-40	90-50
**Average error**	9.1740	9.1544	9.1620	9.1525	9.1875

**Table 6:** Comparitive study in terms of accuracy.

References	3D - System	
	Positioning Algorithm	Error Distance
[16]	Geometrical relationship	0.25–50 cm
[17]	FDM and TDOA	0.020 mm
[18]	Lateration	3 cm
[19]	Genetic algorithm	2–5 cm
This work	Three neural networks	0.4 mm
